# Qualitative Methods for the Inverse Obstacle Problem: A Comparison on Experimental Data

**DOI:** 10.3390/jimaging5040047

**Published:** 2019-04-10

**Authors:** Martina T. Bevacqua, Roberta Palmeri

**Affiliations:** 1 DIIES, Department of Information Engineering, Infrastructures and Sustainable Energy, Università Mediterranea di Reggio Calabria, via Graziella, Loc. Feo di Vito, 89124 Reggio Calabria, Italy; 2 CNR-IREA, National Research Council of Italy, Institute of Electromagnetic Sensing of the Environment, via Diocleziano 328, 80124 Napoli, Italy

**Keywords:** inverse obstacles problem, inverse source problem, joint sparsity, linear sampling method, microwave imaging, orthogonality sampling method

## Abstract

Qualitative methods are widely used for the solution of inverse obstacle problems. They allow one to retrieve the morphological properties of the unknown targets from the scattered field by avoiding dealing with the problem in its full non-linearity and considering a simplified mathematical model with a lower computational burden. Very many qualitative approaches have been proposed in the literature. In this paper, a comparison is performed in terms of performance amongst three different qualitative methods, i.e., the linear sampling method, the orthogonality sampling method, and a recently introduced method based on joint sparsity and equivalence principles. In particular, the analysis is focused on the inversion of experimental data and considers a wide range of (distinct) working frequencies and different kinds of scattering experiments.

## 1. Introduction

The inverse scattering problem aims to retrieve the electromagnetic properties of unknown targets by processing the field they scatter [1,2]. This problem can be of interest in biomedical imaging in order to discriminate between healthy and malignant biological tissues, identify anomalies inside the human body, and build an electromagnetic model of a specific anatomical district [3,4,5]. Moreover, it is relevant in other applications, such as underground prospecting as well as non-destructive testing of materials, in order to detect and characterize buried targets and internal defects [6,7]. 

Due to the non-linearity and ill-posedness of this class of inverse problems [1,8], the quest to develop reliable, accurate, and effective solution methods is still ongoing. As witnessed by many papers published in literature, several researchers and scholars are very active on this topic, covering its challenging computational, algorithmic, modeling, and experimental aspects.

Due to the mathematical difficulties of the problem, many studies have been focused on the introduction of solution methods for the corresponding inverse obstacle problem, whose scope is to recover just the morphological information about the targets, namely their supports, by giving up their electromagnetic properties [9,10,11,12,13,14,15,16]. Such methods are known as qualitative methods and are different from quantitative methods, which aim at retrieving both electrical and morphological properties of the region of interest [2,9]. Indeed, they tackle the problem in its full non-linearity, without involving any approximation and allowing for a widening of the class of retrievable objects; unfortunately, such methods are characterized by a higher computational burden and longer processing time [2]. On the other hand, qualitative methods are characterized by simplified mathematical models and a lower computational burden [9].

Among qualitative methods, it is worth mentioning sampling methods that include the linear sampling method (LSM) [12] (and the related factorization method [13]), as well as the orthogonality sampling method (OSM) [14]. The main idea of these methods is that of sampling the investigation domain in an arbitrary grid of points and computing at each point an indicator function whose energy will assume different values depending on whether the sampled point belongs or not to the scatter. Although both LSM and OSM belong to the class of sampling methods, they exhibit different features. For instance, the LSM indicator function is computed by solving an auxiliary linear problem whose solution involves the adoption of a regularization technique. In a different fashion, the OSM does not require the solution of a linear problem since the indicator function is just related to the evaluation of a scalar product. Moreover, the OSM seems to be more flexible with respect to the measurement configurations.

By taking advantage of recent results in the area of compressive sensing and sparsity promoting techniques [17], a new qualitative method has been very recently introduced in the literature that, provided some conditions hold true, is able to retrieve the boundary of unknown targets rather than their support [15,16]. Such a method exploits the equivalence theorem in the case of dielectric obstacles and takes advantage of the particular distribution assumed by the induced currents in case of perfect electric conductors [18]. Differently from sampling methods, it does not sample the investigation domain in a grid of points, and it does not require the selection of a fixed threshold to discriminate between points inside and outside the targets. However, if compared with LSM and OSM, it shows a heavier computational burden.

Encouraged by the remarkable properties of the methods discussed above, in the following, some comparisons and performance analysis are provided. In particular, the tests are performed against experimental data [19] and consider a wide range of working frequencies and different kinds of (monochromatic) data. 

The paper is organized as follows. In Section 2, the inverse scattering problem is formulated. Moreover, a brief summary of the three considered qualitative methods is given, including a discussion on their expected advantages and disadvantages. In Section 3, some examples considering experimental Fresnel data are reported. Finally, conclusions follow. Throughout the paper we consider the canonical 2D scalar problem (transverse magnetic (TM) polarized fields) and we assume and drop the time harmonic factor $exp\left\{j\omega t\right\}$.

## 2. Materials and Methods

### 2.1. Mathematical Formulation of the Problem

Let D denote the region being tested where targets with support Σ are located. Let $\chi \left(r\right)\;=\;\epsilon_{s}\left(r\right)/\epsilon_{b}\;-\;1$ be the contrast function that relates the electromagnetic properties of the scatterers to those of the host medium, wherein $\epsilon_{s}$ and $\epsilon_{b}$ are the complex permittivities of the scatterers and the background medium, respectively. Let $E_{s}{}^{\infty }\left({\hat{r}}_{m},\;{\hat{r}}_{t}\right)$ be the scattered far-field pattern measured on a closed circle curve Γ in the far zone of the scatterers in direction ${\hat{r}}_{m}$ when a unit amplitude plane wave impinges from direction $\;{\hat{r}}_{t}.$ Then, the equations describing the scattering problem can be expressed as [1]:

(1) $$E_{s}{}^{\infty }\left({\hat{r}}_{m},\;{\hat{r}}_{t}\right)=\underset{D}{\int }G_{b}{}^{\infty }\left({\hat{r}}_{m},\;r\right)W\left(r^{\prime },{\hat{r}}_{t}\right)dr^{\prime }=A_{e}\left[W\right]$$

(2) $$E\left(r,{\hat{r}}_{t}\right)=E_{i}\left(r,{\hat{r}}_{t}\right)+\underset{D}{\int }G_{b}\left(r’,\;r\right)W\left(r^{\prime },{\hat{r}}_{t}\right)dr^{\prime }=E_{i}+A_{i}\left[W\right]$$

where $E_{i}$ and  E are the incident and total field, respectively, W=χE is the contrast source, and $r=\left(x,y\right)$ scans the investigation domain D. Moreover, $G_{b}{}^{\infty }$ is the Green’s function in the far-field zone pertaining to the background medium. Finally,$\;A_{e}$ and $A_{i}$ are short notation for the integral radiation operators. 

In the inverse obstacle problem, one aims at retrieving the support Σ of the target, by giving up its electromagnetic properties, from the (noise corrupted) measured data $E_{s}{}^{\infty }$ when the targets are illuminated by a known incident field $E_{i}$ [9]. Such a problem is non-linear as well as ill-posed because of the properties of the involved operator $A_{e}$ [1,8].

### 2.2. Linear Sampling Method

The LSM is one of the most popular qualitative methods to retrieve objects’ support from the measurements of the corresponding scattered field data [12]. More in detail, it consists in sampling the region under test into an arbitrary grid of points and solving for each point the so-called *far field integral equation* given by:

(3) $$\int_{\Gamma }E_{s}{}^{\infty }\left({\hat{r}}_{m},{\hat{r}}_{t}\right)\;\xi \left(r_{s},{\hat{r}}_{t}\right)\;d{\hat{r}}_{t}=G_{b}{}^{\infty }\left({\hat{r}}_{m},\;r_{s}\right)$$

wherein ξ represents the unknown function and $r_{s}$ the sampling point. Despite the linearity of Equation (3), the evaluation of the solution ξ is not straightforward due to the compactness of the operator at the left hand side, which implies ill-posedness in its inversion [12,20]. Hence, a regularization technique is required. An effective choice is that of solving it by adopting the Tikhonov regularization [20]. Then, the estimation of the unknown support is pursued by evaluating the $l_{2}$ norm (i.e., the “energy”) of the unknown function ξ with respect to $r_{t}$, given by:

(4) $$I_{LSM}\left(r_{s}\right)=\|\xi {\left(r_{s},{\hat{r}}_{t}\right)\|}_{2}^{2}=\int_{\Gamma }{\left|\xi \left(r_{s},{\hat{r}}_{t}\right)\right|}^{2}d{\hat{r}}_{t}$$

The above defined indicator depends on the sampling point $r_{s}$ and assumes a finite value when the sampling point belongs to the unknown object, while it blows up elsewhere [12]. As such, by selecting a fixed threshold, the indicator described in Equation (4) allows to discriminate between points inside and outside the scatterers and finally retrieve Σ [20]. It is worth noting that the computational burden is low, as the solution of Equation (4) just involves the evaluation of the singular values decomposition (SVD) of the measured data [20].

### 2.3. Orthogonality Sampling Method

The OSM has recently been introduced in the literature [14]. It consists in computing the reduced scattered field from the far-field pattern $E_{s}{}^{\infty }\left(r_{m},r_{t}\right)$. Such a reduced field can be computed by evaluating the scalar product between the far field and the Green’s function in far-field zone, that is [14]:

(5) $$E_{s}{}^{red}\left(r_{s},{\hat{r}}_{t}\right)=\int_{\Gamma }E_{s}{}^{\infty }\left({\hat{r}}_{m},{\hat{r}}_{t}\right)\;e^{jk_{b}\;r_{s}\cdot {\hat{r}}_{m}}\;d{\hat{r}}_{m}=\frac{1}{\gamma }{\langle E_{s}{}^{\infty },G_{b}{}^{\infty }\rangle }_{\Gamma }$$

where 〈·,·〉 denotes the scalar product and γ is a constant for a fixed frequency [14]. In a nutshell, Equation (5) tests the orthogonality relation between the far-field pattern and the Green’s function in the far-field zone (apart from a constant). Then, the OSM indicator $I_{OSM}\left(r_{s}\right)$ is defined as:

(6) $$I_{OSM}\left(r_{s}\right)=E_{s}{}^{red}{\left\|r_{s},{\hat{r}}_{t}\right\|}_{2}^{2}=\int_{\Gamma }{\left|E_{s}{}^{red}\left(r_{s},{\hat{r}}_{t}\right)\right|}^{2}d{\hat{r}}_{t}$$

and achieves large values when the far-field pattern approaches the one for the background Green’s function for the considered sampling point [14]. Again, by selecting a fixed threshold, it is possible to discriminate sampling points belonging to the target support [14].

### 2.4. Shape Reconstruction Via Joint Sparsity Based Inverse Source and Equivalence Principles

In order to deal with inverse obstacle problems, one can also take advantage of the joint solution of a number of related inverse source problems that consist of recovering the induced currents from the knowledge of the measured far field pattern $E_{s}{}^{\infty }$ [15,16]. The strategy is obviously suggested by the fact that the support of W is exactly equal to Σ whatever incident field $E_{i}$ is. Note that inverse source problems are linear (see Equation (1)) but are still (severely) ill-posed such that some additional care is needed. 

In this respect, note that when a generic electromagnetic field is propagating in the space in the presence of dielectric obstacles, it induces some currents inside the obstacles, which are expected to be different from zero inside Σ and in turn become sources of the scattered field data. However, by virtue of the equivalence theorem [18] (which is a key point of the approach), the measured scattered field can be eventually conceived as the one radiated by some equivalent surface currents, i.e., electric $W_{s}$ and magnetic $W_{smx}$ and $W_{smy}$ surface currents, which can play the role of the original ones. 

The main advantage of considering the equivalent currents rather than the original ones is that they are distributed over the boundary of Σ. As a consequence, they can be identified by only a few non-zero pixels in the investigation domain and, hence, can be considered sparse with respect to the pixel basis representation. Then, a possible idea for their retrieval is that of looking for the sparsest current distributions, which contemporarily satisfy the measured data. To this end, sparsity promoting approaches can be exploited [17]. 

Interestingly, the property of sparsity displayed by the equivalent currents holds true regardless of the direction of illumination ${\hat{r}}_{t}$ or the kind of measurement configuration. In order to enforce this joint sparsity among the different scattering experiments, an auxiliary variable B can be defined as the common upper bound on the amplitude of the equivalent currents for the different scattering illumination conditions. Accordingly, the problem is recast as [15,16]:

(7a) $$\min{\left\|B(r)\right\|}_{1}$$

(7b) $$s.t.\;\;\;\;\;{\left\|E_{s}{}^{\infty }-A_{e}\left[W_{s}\right]-A_{mx}\left[W_{smx}\right]-A_{my}\left[W_{smy}\right]\right\|}_{2}\leq \delta$$

(7c) $$\begin{array}{c} \left|W_{s}\left(r,{\hat{r}}_{t}\right)\right|\leq B\left(r\right)\\ \left|W_{smx}\left(r,{\hat{r}}_{t}\right)\right|\leq B\left(r\right),\;\;\;\;\forall \;{\hat{r}}_{t}\in \Gamma \\ \left|W_{smy}\left(r,{\hat{r}}_{t}\right)\right|\leq B\left(r\right) \end{array}$$

where ${\left\|\cdot \right\|}_{1}$ is the $l_{1}$ norm, and $A_{mx}$ and $A_{my}$ are the integral radiation operators that relate the magnetic surface currents to the scattered electric fields.

The variable B does not depend on the direction of illumination ${\hat{r}}_{t}$, but only on the position r in the investigation domain. Moreover, it is expected to assume non-zero values only on the boundary of Σ such that it will act as a “boundary” indicator. For this reason, the method is called *boundary retrieval through inverse source and sparsity* (B-IS). As a consequence, differently from LSM and OSM, there is no need for a thresholding to retrieve the support from the indicator map, as the approach directly retrieves the boundary of the targets.

Note the approach can be also applied to the case of perfect electrical conductors. In fact, in such a case, the induced currents W exist only on the boundary of Σ and there is no need for introducing equivalent surface currents. Then, the optimization problem (7) is further simplified [15,16]. 

As a final comment, note that the B-IS method belongs to the class of the joint sparsity-promoting procedures. In particular, the joint sparsity used herein is not enforced by means of the mixed norm $l_{1}-l_{2}$ as in References [21,22], but through the definition of the auxiliary variable B. 

### 2.5. Applicability and Limititations of the Three Methods 

In LSM, the far field Equation (3) can be interpreted as an attempt to synthetize induced currents such that their radiating part is focused and/or circularly symmetric with respect to the given sampling point [20,23]. As such, it correctly works as long as the object is convex and the non-radiating component of the induced currents is negligible [20]. Usually, when the product of the maximum amplitude of the contrast χ times the maximum size of the target is large, which corresponds to an increasing amount of non-radiating sources, it does not guarantee reliable reconstructions. 

The OSM is based on a test of orthogonality, which allows one to compute the reduced scattered field. As far as its physical interpretation is concerned, it has been recently argued in Reference [24] that the reduced scattered field is related to the radiative part of the currents. As such, similarly to LSM, the OSM indicator does not take into account the non-radiating part of the currents, so that, again, limitations are expected with increasing electrical dimensions of the scatterer. 

While both the LSM and OSM act on volumetric currents, which are located on all points belonging to the support of the target, B-IS aims at jointly solving several inverse source problems, wherein one is looking for currents that, under some reasonable hypotheses, are located on the boundary of Σ. Notably, the optimization problem (7) does not aim at retrieving exactly the equivalent currents but rather to find a common upper bound to their amplitudes. Also note the possible (superficial) non-radiating currents do not seem to play any role in the process, while the possible non-radiating volumetric currents are filtered out by the sparsity regularization. For all the above, the B-IS method can be understood as more robust with respect to the presence of non-radiating currents than LSM and OSM. Moreover, the upper bound in Equation (7) can be understood as a trade-off between sparsity promoting and data fitting. As a consequence, B-IS will be able to retrieve at least the convex-hull of the targets. Remarkably, probably because of the use of the $l_{1}$ norm rather than the $l_{0}$ pseudo-norm, one can also be able to retrieve the shape of some non-convex target [15]. As a consequence of all the above, B-IS is expected to fail when the boundary of the target is not sparse (for instance in case of a ragged boundary), or when, according to the sparsity theory [17], the number of measurements is not sufficiently large with respect to the degree of sparsity of the target contour. 

Concerning the requirements about the kind of measured data, the LSM needs to correctly discretize the integral operator in Equation (3) by collecting a sufficient number of data in multiview-multistatic measurement configuration. As a consequence, even if the product of the maximum amplitude of the contrast χ times the maximum size of the target is not large, the undersampling of the measured data can compromise the reconstructions. On the other side, the OSM requires an accurate discretization of the integral in Equation (5) over the measurements curve, and hence, a sufficient number of measurements. However, differently from LSM, OSM can compensate the low number of data points by considering other kind of experiments different from multiview-multistatic ones. In fact, OSM and B-IS exhibit wide flexibility with respect to processed data and can offer the possibility of combining information arising from frequency diversity without the need of a posteriori merging the single results. To this end, the OSM indicator can be easily modified by integrating over a given frequency band [14,24], and the B-IS indicator function implicitly defined by Equation (7c) can be redefined as the upper bound for the different sources at the different frequencies [15].

Another important difference among the methods regards the computational burden. The OSM indicator does not involve the solution of an ill-posed problem but only the evaluation of a scalar product. This implies more robustness with respect to measurement errors on the scattered data and a very low computation burden. On the contrary, the LSM indicator function is computed by solving an auxiliary linear problem, whose solution involves the adoption of a regularization technique. However, the main computational effort of the method is the evaluation of the SVD of measured data, whose dimension is only dictated by the number of scattering experiments. This task has to be carried out only once, since the kernel of the linear system is the same for all sampling points. With respect to the LSM and the OSM, B-IS exhibits a heavier computational burden, which drastically increases with the number of unknowns, since an $l_{1}$ norm optimization problem has to be solved. On the other side, the problem underlying the B-IS belongs to the class of convex programming problems, whose single optimal solution can be reached using any common local optimization procedure. 

In terms of reconstruction accuracy, both the LSM and OSM require the selection of a fixed threshold to discriminate between points inside and outside the targets. In a different fashion, the B-IS does not provide an indicator map but directly looks for the boundary of the target. As such, it is expected to be more accurate. 

These aspects, which are briefly summarized in Table 1, will be further investigated in the following numerical section.

## 3. Results

In this section, an accurate comparative study was performed against the experimental data set provided by the Institute Fresnel of Marseille [19], typically adopted to benchmark inverse scattering procedures. In particular, four targets from the 2001 Fresnel database were considered, i.e.,:

- the DielTM target, which consists of a dielectric homogeneous cylinder of radius 1.5 cm and relative permittivity 3 ± 0.3
- the RectTM_Dece target, which is a rectangular metallic target of 25.4 mm × 12.7 mm not centered with respect to the azimuthal positioner axis;
- the U-TM shaped target, which is a metallic U-shaped target with dimension 80 × 50 mm^2^
- the TwinDielTM target, which consists of two identical dielectric homogeneous cylinders of radius 1.5 cm and relative permittivity 3 ± 0.3.

**Figure 1:**
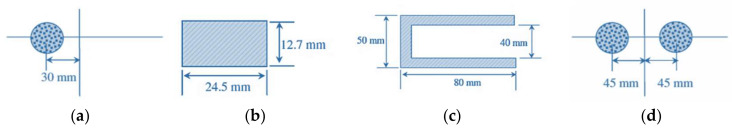
Targets from 2001 Fresnel dataset: (**a**) DielTM target; (**b**) RectTM_Dece target; (**c**) U-TM-shaped target, and (**d**) TwinDielTM target.

The complete description of the targets and the measurement set-up can be found in Reference [19], while a schematic sketch is shown in Figure 1. The data were collected under a partially aspect-limited configuration, where primary sources completely surrounded the targets, but for each illumination, the measurements were taken only on an angular sector of 240° achieved by excluding the 120° sector centered on the incidence direction. 

For the dielectric targets, the working frequencies could change among the interval 1–8 GHz with a step of 1 GHz. Also, 12 GHz and 16 GHz data were available. On the other side, for the metallic targets the working frequencies can change among the interval 2–16 GHz with a step of 2 GHz. For the sake of brevity, only some significant reconstructions corresponding to some frequencies will be shown in the following. Moreover, for each target, two different datasets have been considered. The first one consisted of a 36 × 36 multiview-multistatic data matrix (the number of measurements and views M is equal to 36), while the second one corresponded to a data matrix with halved dimensions, that is M equal to 18. The investigated area of 0.1775 m × 0.1775 m was discretized in 40 × 40 cells. 

In the following examples the single frequency indicators were normalized with respect to their maximum values. In particular, the LSM indicator were rescaled as described in Reference [25]. 

Note that the number of independent data were lower with respect to the numbers of unknowns. This is a common problem of all inverse scattering and inverse obstacles problems. In fact, one is not able to collect data at will, as only a finite number of independent experiments can be performed [26]. However, the qualitative methods we were comparing did not look for a quantitative reconstruction in every pixel of the scenario, but they looked for some qualitative behavior of the indicator. Moreover, all three methods somehow exploited additional regularizations. 

### 3.1. Convex Dielectric Target

**Figure 2:**
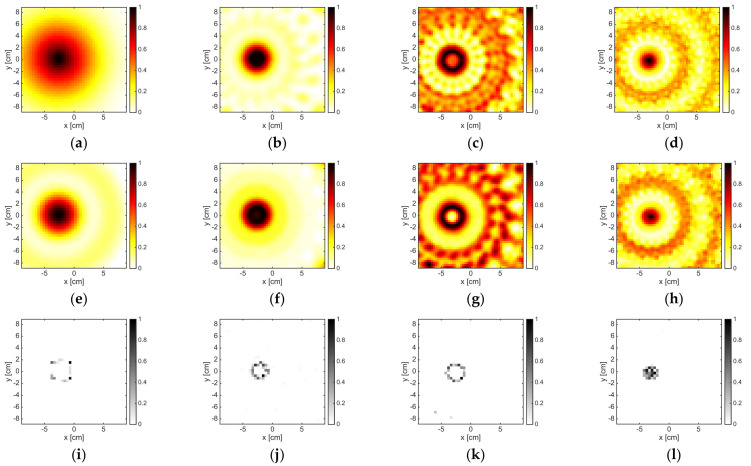
The DielTM target. From left to right: 2 GHz, 6 GHz, 12 GHz, and 16 GHz. From top to bottom: LSM, OSM, and B-IS indicators. The number of measurements and views was M = 36.

The results corresponding to the single dielectric circular cylinder are reported in Figure 2. As can be seen, at the lowest frequencies, the three methods could localize the cylinder (whose diameter was 0.1$\lambda_{b}$ at 1 GHz and 0.2$\lambda_{b}$ at 2 GHz ($\lambda_{b}$ was the wavelength in free space)). Its size was overestimated, especially by LSM and OSM, while instead the B-IS method was not able to correctly identify its shape. In the frequency range of 3–8 GHz, the performance of the three methods improved and the support was correctly retrieved. This circumstance also held true at higher frequencies, although some artifacts and ambiguities started to emerge. For instance, at 12 GHz, the OSM indicator was characterized by a hole at the center of the cylinder, while at 16 GHz, some artifacts appeared in the background. On the other hand, the B-IS indicator wrongly suggested some pixels inside the target, but such a circumstance did not compromise the correct estimation of the target support.

**Figure 3:**
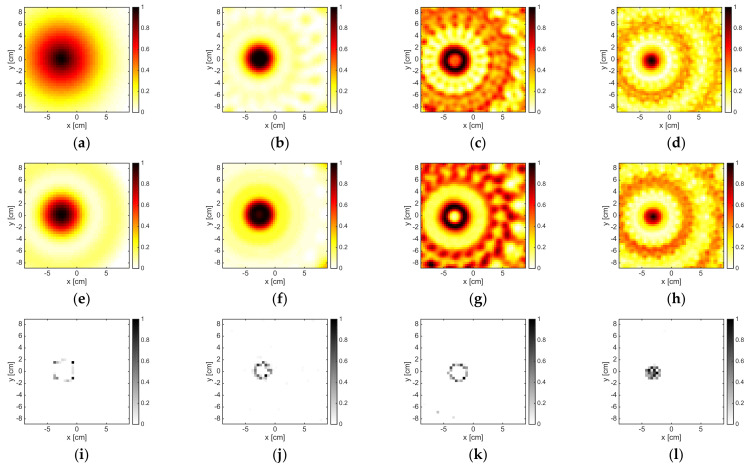
The DielTM target. From left to right: 2 GHz, 6 GHz, 12 GHz, and 16 GHz. From top to bottom: LSM, OSM, and B-IS indicators. The number of measurements and views was M = 18.

When a reduced amount of data was processed, both OSM and LSM did not work fine at the frequencies of 12 and 16 GHz, as shown in Figure 3. This was due to the fact that the amount of data, which was needed in order to correctly discretize the far field Equation (3), as well as to correctly compute the reduced scattered field Equation (5), was larger than M, whereas the size of the target became large in terms of wavelength. The B-IS was instead robust with respect to the reduction of data. In fact, the same performance (with respect to the case M = 36) was guaranteed when M = 18. 

In order to quantitatively assess the quality of the reconstructions, the following dimensional error $err_{\Delta }$ was evaluated:

(8) $$err_{\Delta }=\frac{r_{rec}-r_{ref}}{r_{ref}}$$

where $r_{rec}$ and $r_{ref}$ being the radius of the reconstructed and the reference cylinders, respectively. This metric allows one to appreciate the performance of the method in reconstructing the size of the target. In evaluating these errors, two different methods have been adopted to binarize the LSM and OSM indicator map. As the indicator maps are continuous functions, we first binarized them by applying the Canny edge detector [27,28] to its dB plot and set it to one of the pixels internal to the identified contour (see Table 2). The second method instead consisted of replacing all pixels in the input image with value greater than a given level $L\;\in \;]0,1]$ with the value 1 and replaced all other pixels with the value 0 (see Table 3). Note that the B-IS method did not require the adoption of a binarization technique. By comparing Table 2 and Table 3, one can conclude that:

- the B-IS was more accurate in estimating the radius of the cylinder and it was robust with respect to the reduction of M;
- the choice of the thresholding techniques could impact the reconstruction of the support of the target. Indeed, the dimensional errors in Table 2 were different from the one in Table 3. In particular, the Canny edge detector-based approach led to an overestimation of the radius of the cylinder, while the second approach (L = 0.8) implied more accurate estimations, as witnessed by the lower dimensional errors. Notably, the accuracy of the reconstructions when LSM and OSM were adopted strongly depended on how the indicator map was binarized and on the adopted threshold.

**Figure 4:**
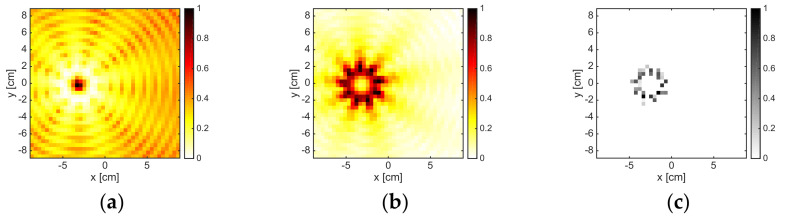
The DielTM target at 12 GHz. From left to right: LSM, OSM, and B-IS indicators. The dimension of the data matrix was 72 × 9.

Finally, in order to emphasize the flexibility of OSM and B-IS with respect to the measurement configuration, the inversion of a 72 × 9 data matrix was performed. In such a case the number of experiments was reduced to 9, while the measurements were increased to 72. The corresponding indicators are reported in Figure 4. B-IS was able to quite accurately retrieve the target support, while LSM only localized the target. Finally, differently from LSM, OSM was able to provide a rough reconstruction of the support, as it was sensitive with respect to the number of measurements rather than to the number of experiments. 

### 3.2. Convex Metallic Target

**Figure 5:**
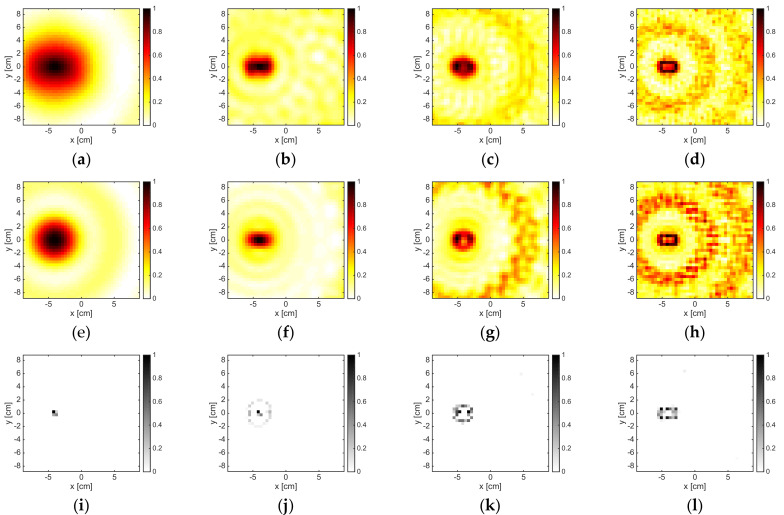
The RectTM_Dece target. From left to right: 2 GHz, 6 GHz, 10 GHz, and 16 GHz. From top to bottom: LSM, OSM, and B-IS indicators. The number of measurements and views was M = 18.

Figure 5 depicts the reconstructions corresponding to the metallic rectangular targets pertaining to 2, 6, 10, and 16 GHz and M = 18. As can be observed, at the lowest frequency, the methods were just able to localize the scatterer, especially the B-IS method, which identified only some pixels in the center of the target. In fact, at 2 GHz, the target was just 0.2$\lambda_{b}$ × 0.1$\lambda_{b}$ large. By increasing the frequency, the capability of the methods in retrieving the shape of the metallic target improved. For instance, at 6 GHz, both LSM and OSM correctly retrieved the unknown support, while B-IS overestimated its vertical dimension. However, in the frequency range of 8–16 GHz, both LSM and OSM came to exhibit some artifacts and did not retrieve an indicator map that was monotonically decreasing outside the actual support of the scatterer. This was correlated to the fact that the electromagnetic size of the target became larger and the amount of data needed to correctly solve Equation (3), as well as compute the reduced field Equation (5), was much larger than M. Indeed, by considering M = 36, reliable results, which are not shown for the sake of brevity, could be achieved. Notably, when M = 18, the B-IS was still able to reconstruct the actual support of the rectangular object also in the range of 8–16 GHz, as shown in Figure 5k,l.

### 3.3. Non-Convex Metallic Target

**Figure 6:**
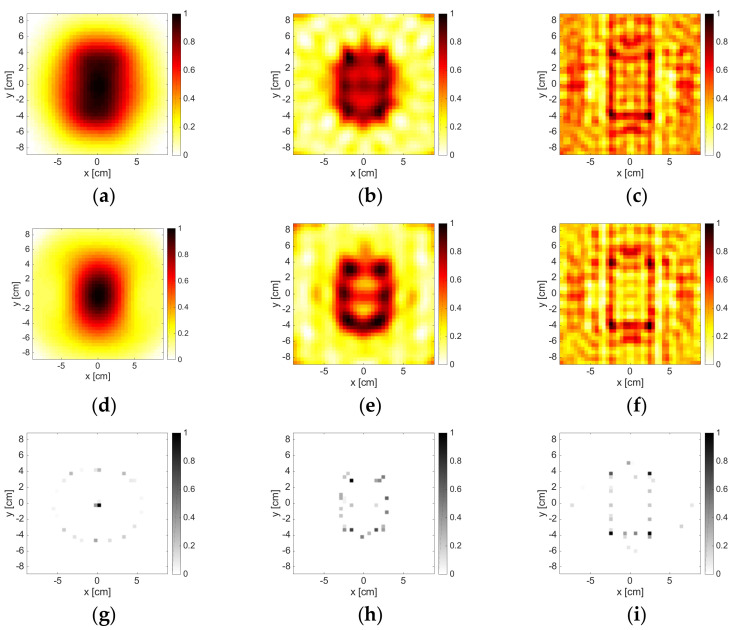
The U-TM-shaped target. From left to right: 2 GHz, 6 GHz, and 10 GHz. From top to bottom: LSM, OSM, and B-IS indicators. The number of measurements and views was M = 18.

The same behavior observed in the previous examples also holds true in case of the U-TM-shaped target (see Figure 6). In fact, at 2 GHz, the target was detected by all three methods and approximately localized by LSM and OSM, but only its convex-hull was retrieved by LSM and OSM. On the other hand, at higher frequencies, the support was only approximately retrieved by all three methods. In particular, some artifacts were present due to the mutual interactions between the “arms” of the target, especially in the LSM and OSM indicators, which were sensitive to the non-radiating part of the currents. Note that such a target belongs to the class of non-convex targets, so that it represents a more challenging case with respect to the previous ones. 

**Figure 7:**
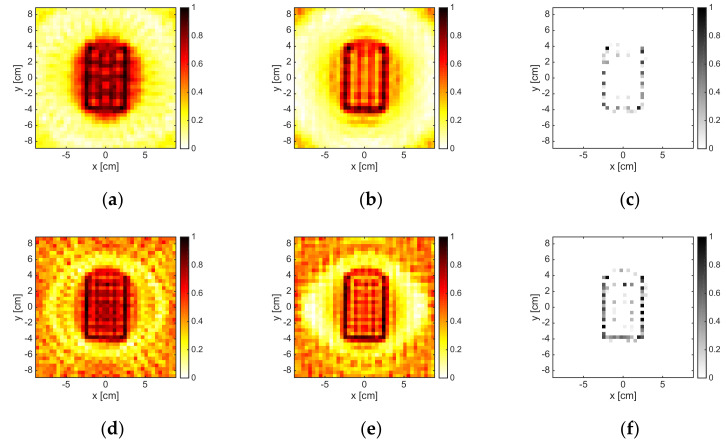
The U-TM-shaped target. From top to bottom: 12 GHz and 16 GHz. From left to right: LSM, OSM, and B-IS indicators. The number of measurements and views was 36.

No significant reconstructions were obtained by means of the three methods for M = 18 and a working frequency belonging to 12–16 GHz. In case of M = 36, the reconstructions are shown in Figure 7. As it can be seen, B-IS correctly retrieved the support of the target, apart from some spurious pixels, while the LSM did not provide reliable results due to the relevant presence of non-radiating currents and a high degree of non-linearity. Finally, the OSM indicator provided a rough support estimation only at 12 GHz.

### 3.4. Multiple Dielectric Targets

**Figure 8:**
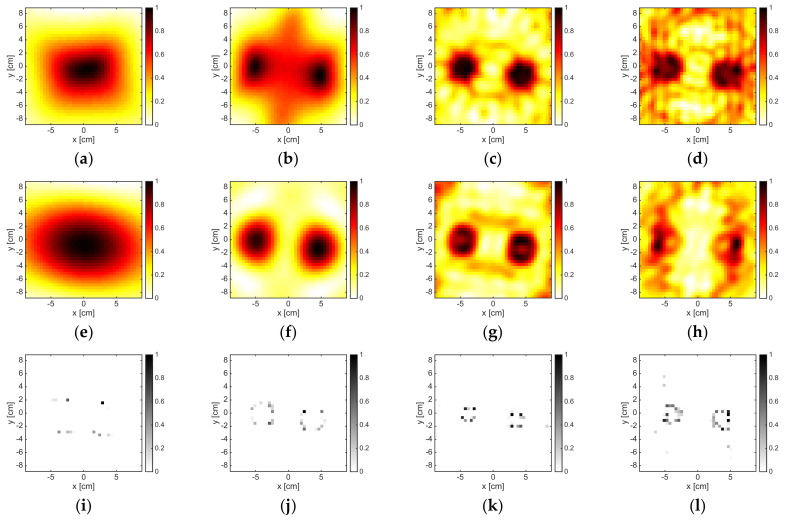
The TwinDielTM target. From left to right: 1 GHz, 2 GHz, 6 GHz, and 8 GHz. From top to bottom: LSM, OSM, and B-IS indicators. The number of measurements and views was M = 18.

The results pertaining to the two identical cylinders are shown in Figure 8 for a number of experiments and measurements corresponding to M = 18. At 1 GHz, the methods could only detect the presence of an obstacle, but they could not identify the presence of two disjoint objects. On the contrary, at 2GHz, it was possible to distinguish two different targets; in particular, from the OSM map, one could also understand their shape. Note that the distance between the targets was 60 mm, which corresponded to 0.2$\lambda_{b}$ at 1 GHz and 0.4$\lambda_{b}$ at 2 GHz. When the frequency increased, both LSM and OSM worked fine. However, when the frequency was 6 GHz, some undesired artifacts occurred.

**Figure 9:**
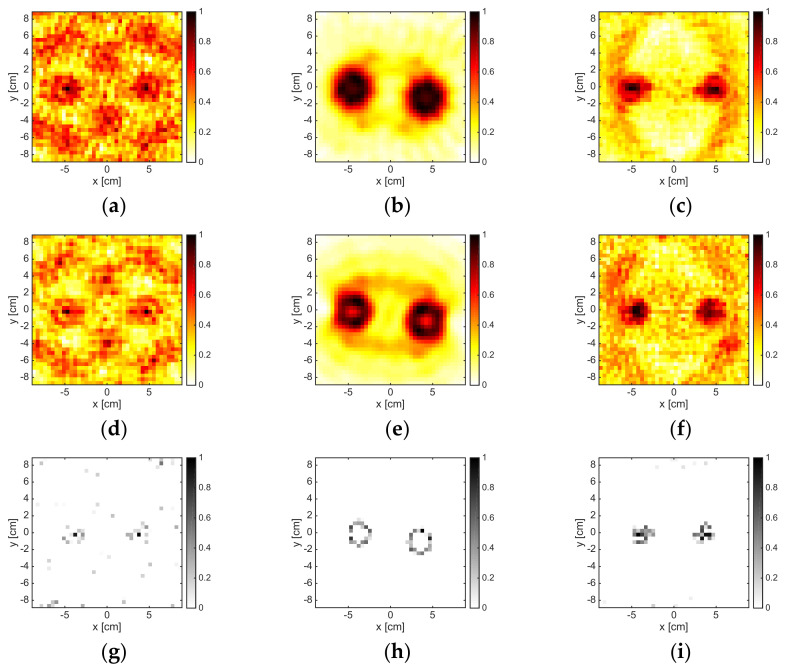
The TwinDielTM target. From left to right: 16 GHz (M = 18), 8 GHz (M = 36), and 16 GHz (M = 36). From top to bottom: LSM, OSM, and boundary indicators. The number of measurements and views was M = 18 for the first column and M = 36 for the last two columns.

On the contrary, the B-IS method is able to provide quite accurate reconstructions of the support also in the frequency range 6–8 GHz. When the frequency and the size of the overall target’s system were further increased, completely meaningless reconstructions were obtained by LSM and OSM, while B-IS indicator at least localized the two targets (see Figure 9g). In order to improve the reconstruction accuracy of the three methods, it was necessary to process a higher amount of data. This was witnessed by the reconstructions reported in Figure 9 corresponding to M = 36 at both 8 GHz and 16 GHz. However, at 16 GHz, the increase of the number of data was not enough for LSM and OSM to provide indicator maps free of artifacts.

## 4. Conclusions

The paper contributes to understand and discuss some strong and weak points of three qualitative methods for the inverse obstacle problem. In particular, a comparison among the popular LSM, the OSM, and the more recent B-IS was reported by considering the processing of the 2001 Fresnel dataset, which included both dielectric and metallic targets, as well as non-convex and multiple targets. Different frequencies and amount of data were considered for each method. In all cases, because of the characteristics of the Fresnel measurements setup, data were aspect-limited, i.e., not all incidence observation angle couples were available (for instance the monostatic data was missing). Also, scattered field data turned out to be undersampled at the higher frequencies with respect to the criteria (depending on the dimension of the investigation domain) discussed in Reference [26].

Results suggested that at lower frequencies, reliable reconstructions were guaranteed if the size of the target was higher than 0.2$\lambda_{b}$. Indeed, for lower dimensions, the three methods could only localize the target, while its size was overestimated. Such a result was fully consistent with expected limitations on resolution implied by the diffraction theorem [29] and the results in Reference [26]. When the frequency was much larger, LSM and OSM began to exhibit some artifacts and, if the amount of data was not sufficient or due to the presence of non-negligible non-radiating sources, they could provide unreliable reconstructions. Notably, the B-IS seemed to be more robust with respect to LSM and OSM when undersampling data and/or increasing the frequency. In fact, it could still provide accurate indicator maps in a number of cases where LSM and OSM failed. 

The examples have also proved that both OSM and LSM were sensitive to the choice of the binarization technique to discriminate between points inside and outside the scatterer. On the other hand, both OSM and LSM had a negligible computational burden, while B-IS had to solve a CP problem whose computational burden increased with the cells used to discretize D, as well as with M. In the considered examples, the computational time amounted to a few seconds for OSM and LSM, and to a few minutes for B-IS. 

Future work will be devoted to extending such a performance comparison to other qualitative methods, for instance, the modified version of LSM [30,31].

## Figures and Tables

**Table 1:** Summary of strong and weak points of the three solution methods.

	LSM	OSM	B-IS
**Reconstruction accuracy**	medium	medium	high
**Computational burden**	low	very low	high
**Flexibility with respect to the kind of data**	low	high	high

**Table 2:** Dimensional errors for the DielTM target. LSM and OSM indicators were binarized by applying the Canny edge detector. The symbol * indicates the cases wherein the indicator maps were not reliable, and the dimensional error was not computed.

Freq (GHz)	LSM		OSM		B-IS	
	M = 36	M = 18	M = 36	M = 18	M = 36	M = 18
2	1.4	0.47	0.97	0.97	0.18	0.18
6	0.55	0.55	0.69	0.74	0.18	0.11
12	1	*	1	*	0.18	0.18
16	1	*	0.18	*	0.18	0.11

**Table 3:** Dimensional errors for the DielTM target. LSM and OSM indicators were binarized by selecting L = 0.8. The symbol * indicates the cases wherein the indicator maps were not reliable, and the dimensional error was not computed.

Freq (GHz)	LSM		OSM		B-IS	
	M = 36	M = 18	M = 36	M = 18	M = 36	M = 18
2	0.37	0.32	0.11	0.11	0.18	0.18
6	0.22	0.09	0.27	0.28	0.18	0.11
12	0.47	*	0.47	*	0.18	0.18
16	0.03	*	0.47	*	0.18	0.11
